# Essential Skills for Health Communication, Barriers, Facilitators and the Need for Training: Perceptions of Healthcare Professionals from Seven European Countries

**DOI:** 10.3390/healthcare11142058

**Published:** 2023-07-18

**Authors:** Dina Zota, Dimitrios V. Diamantis, Konstantinos Katsas, Pania Karnaki, Thomas Tsiampalis, Piotr Sakowski, Costas A. Christophi, Eleni Ioannidou, Sara Darias-Curvo, Victoria-Luise Batury, Hendrik Berth, Anja Zscheppang, Maike Linke, Sotiris Themistokleous, Afroditi Veloudaki, Athena Linos

**Affiliations:** 1 PROLEPSIS Civil Law Non-Profit Organization of Preventive Environmental and Occupational Medicine, 15121 Athens, Greece; d.zota@prolepsis.gr (D.Z.); k.katsas@prolepsis.gr (K.K.); p.karnaki@prolepsis.gr (P.K.); a.veloudaki@prolepsis.gr (A.V.); a.linos@prolepsis.gr (A.L.); 2 Medical School, National and Kapodistrian University of Athens, 11527 Athens, Greece; 3 Department of Nutrition and Dietetics, Harokopio University, 17676 Athens, Greece; ttsiampalis@gmail.com; 4 SAQDATA, 92-637 Lodz, Poland; piotr.r.sakowski@gmail.com; 5 Cyprus International Institute for Environmental and Public Health, Cyprus University of Technology, Limassol 3041, Cyprus; costas.christophi@cut.ac.cy (C.A.C.); eleni.ioannidou@cut.ac.cy (E.I.); 6 Centro de Estudios de Desigualdad Social y Gobernanza, University of La Laguna, 38200 Santa Cruz de Tenerife, Spain; sadacur@ull.edu.es; 7 Research Group Medical Psychology and Medical Sociology, Division of Psychological and Social Medicine and Developmental Neurosciences, Technische Universitaet Dresden, D-01307 Dresden, Germany; victoria-luise.batury@uniklinikum-dresden.de (V.-L.B.); hendrik.berth@uniklinikum-dresden.de (H.B.); anja.zscheppang@tu-dresden.de (A.Z.); maike.linke@ukdd.de (M.L.); 8 Center for Social Innovation “CSI”, Nicosia 1010, Cyprus; sotiris@csicy.com

**Keywords:** health communication, healthcare professionals, communication skills, health communication training

## Abstract

Many healthcare professionals are unaware of the necessary skills and barriers hindering interpersonal health communication. This study aimed to evaluate the healthcare professional’s perception regarding health communication training’s necessity, barriers, facilitators and critical skills in health communication. Data from a cross-sectional online survey in the framework of the H-Com project were utilized. The study included 691 healthcare professionals (physicians, nurses, students and allied health professionals) from seven European countries. Only 57% of participants had participated in health communication training, while 88.1% of them indicated a willingness to be trained in health communication. Nurses were more likely (OR = 1.84; 95% CI 1.16, 2.91) to have received such training, compared to physicians. Most examined communication skills, barriers and facilitators of effective communication, and perceived outcomes of successful communication were considered crucial for most participants, although physicians overall seemed to be less concerned. Most agreed perceived outcomes were improved professional–patient relations, patient and professional satisfaction, physical and psychological health amelioration and patients’ trust. Nurses evaluated the importance of these communication skills and communication barriers, facilitators and outcomes higher than physicians. Physicians may underestimate the importance of communication skills more than nurses. Health communication should become an integral part of training for all health professionals.

## 1. Introduction

Interpersonal communication between patients and their doctors is crucial as it can significantly improve prognosis, therapy and patient satisfaction, while reducing healthcare costs [1,2,3]. Effective communication between healthcare professionals and patients can not only strengthen a patient’s relationship with the physician but also ameliorate health outcomes through enhanced compliance with treatment and patient satisfaction [2,3,4,5,6]. In contrast, miscommunication can prevent patients from correctly understanding their discharge status, diagnosis and therapeutic regimen [7].

The importance of yielding communication in successful consultations and patient satisfaction is often underestimated by physicians [2], a disquieting phenomenon, especially when considering that through their career physicians will conduct as many as 150,000 patient consultations [1].

To improve communication between healthcare professionals and their patients, it is important to invest in Health Communication Training (HCT), which should be an indispensable part of the core training of every healthcare professional [8]. According to the definition provided by the Centers for Disease Control and Prevention (CDC) and the U.S. National Cancer Institute, health communication is “the study and use of communication strategies to inform and influence individual decisions that enhance health” and can consist of both written and verbal aspects [9]. Health communication includes a broad spectrum of communication practices, but throughout this study, we will focus on interpersonal patient and healthcare professional communication. 

Health communication between healthcare professionals and patients is influenced by multiple barriers connected mainly to the healthcare professionals’ working environment, such as the pressure of productivity and the lack of experience among medical students or new professionals [10]. Overestimation of communication abilities, common among many physicians, can also lead to poor health communication [11,12]. Another barrier in effective communication is the widespread use of the information-based model, where information about symptoms and treatment options are simply explained to the patient without accounting for their views, treatment comprehension or emotional response [11]. Health communication, in contrast, is based on shared-decision model consultations, which account for the patients’ comprehension and satisfaction pertinent to their therapeutic options [13]. However, such a patient-targeted approach requires that healthcare professionals have the necessary communication skills and are adequately trained in communication principles, which is a significant facilitator for successful health communication [14].

Given the importance of health communication for the doctor–patient relationship, healthcare professionals should devote significant time to HCT, if available, in order to improve their performance in their everyday practice [3,4,8,10,15]. The acknowledgment of the importance of health communication, especially by organizations such as the WHO [16], led to the introduction of relevant training in limited medical and educational programs [3]. As part of the H-Com project [17], the consortium collected and recorded relevant training opportunities in 2016, building an online database of HCT courses in Europe. The study showed that HCT opportunities were available only in a small number of European medical school programs, even though this is expected to have risen slightly in previous years, and HCT in the form of continuous education was scarce [18]. 

The current study explores healthcare professionals’ perceptions of health communication, including the perceived importance of specific communication skills, barriers and facilitators in different healthcare settings. Moreover, the article explores the extent to which health professionals have received HCT and which are the characteristics that may influence the likelihood of having received training in health communication.

## 2. Materials and Methods

### 2.1. Study Design

The H-Com Project was an Erasmus+ co-funded multi-partner initiative aiming to build and strengthen communication skills among healthcare professionals. The project’s objectives were to map the existing HCT opportunities across Europe; explore the needs and perceptions of healthcare professionals concerning communication; and develop, test and evaluate an HCT initiative through the provision of vocational education to healthcare professionals. Within the context of the H-Com Project, a cross-sectional survey was conducted, targeting health professionals (including physicians, nurse practitioners and other allied health professionals). An online questionnaire was developed to elucidate the intricacies of relationships between health professionals and patients, particularly concerning health communication. The purpose-made pilot questionnaire was based on an in-depth review of the literature and the outcomes of a qualitative study on physician–patient communication perceptions, barriers and the training needs of healthcare professionals [19].

The online questionnaire, comprising 35 close-ended questions, included components regarding demographic characteristics, educational experience, professional status, experience with HCT, assessment of the necessity and willingness to participate in HCT and barriers and facilitators of implementing health communication across different healthcare settings. Professionals were asked to rank the importance of various communication skills and practices, potential barriers and facilitators to effective health communication using a Likert scale, with possible answers ranging from 1 (not important at all) to 5 (very important). The questionnaire was developed in English and was then translated by professionals in the field of health communication working in the project’s partner organizations into the consortium languages, i.e., German, Greek, Polish and Spanish.

### 2.2. Setting

The H-Com Consortium Partners represented five European countries, namely Cyprus, Germany, Greece, Poland and Spain. Health professionals in the consortium countries, as well as in other European countries, were invited to participate in the online survey. More specifically, participants were invited to take part between September 2016 and April 2017, during which 750 healthcare professionals in Greece and Cyprus and 1200 contacts in other EU countries from hospitals, universities, medical associations and academics were contacted to fill in the questionnaire. Participants were contacted via email and participation was anonymous. The collected data were stored by the leading organization in data collection (Prolepsis Institute) and all data were password protected. Participants were informed about the data management procedures. 

### 2.3. Study Sample

The original study sample included 743 health professionals. Participants who did not specify health specialty/training or age were excluded from further analysis (*n* = 41; 5.5%). Hence, the final study sample consisted of 702 participants, of whom 691 specified if they had ever received HCT.

### 2.4. Statistical Analysis

Statistical analysis was performed with IBM SPSS statistics, version 23. Qualitative characteristics are presented overall and by profession, as number of observations (*n*) and percentages (%). The differences among professional groups were assessed using the Pearson’s chi-square test. Healthcare professionals’ perceptions are presented as percentages for categorical variables and as mean (standard deviation) for continuous variables. Analysis of variance (ANOVA) was utilized to compare healthcare professionals’ all quantitative variables among professional groups. The Bonferroni correction for post hoc analyses was performed.

The association of healthcare professionals’ characteristics and the likelihood of having received HCT was evaluated through a univariate logistic regression model (all case analysis). A further multivariate logistic regression model was constructed, adjusting for all the confounders. We considered all characteristics that indicated differences between the professional groups as potential confounders for every possible predictor (excluding the examined predictor each time).

## 3. Results

### 3.1. Descriptive Characteristics

The characteristics of the study sample and subgroups according to their professional expertise are presented in Table 1. About 64% of the sample was female. Most participants were employed in Greece (21.2%), while the rest were employed in Italy, Spain, Germany, Poland, Portugal, Cyprus and other countries, including 14 European and 6 non-European countries. The majority were physicians (45%), followed by nurses, other allied health professionals (i.e., psychologists, paramedics, health administration staff, nursing assistants, social workers, dentists/dental hygienists, dietitians/nutritionists, health promotion specialists, public health specialists) and students from health-related sectors. Among students, nurses and allied health professionals, the majority of them were female, as opposed to physicians (*p* < 0.001). Nurses and students seem overall of younger age (<35 years old) compared to physicians and allied health professionals (*p* < 0.001). About two in five physicians reported that they had attained a PhD or equivalent degree, while nurses and allied health professionals reported lower academic achievements than PhD level (*p* < 0.001; 10.3% and 22.6%, respectively).

### 3.2. Perceptions Regarding the Need and Willingness to Participate in HCT

More students and nurses had received HCT in the past (Table 2), compared with allied health professionals and physicians (*p* < 0.001). Most healthcare professionals received HCT during residency training, at the postgraduate level or graduate level, in the form of continuous education training, or other sources. The vast majority of the participants were willing to participate in HCT and about 9 out of 10 recognized that training physicians or nurses on health communication skills is necessary (Table 2).

### 3.3. The Benefits of Effective Communication and the Importance of Communication Skills–Reported Perceptions

More than 97.5% of the participants agreed that successful health communication is predominantly associated with improved physician/nurse–patient relations, followed by increased patient satisfaction (97.4%) (Table 3).

Other beneficial outcomes attributed to effective communication were enhanced patient trust (agreement rate: 96.6%), prescribed medication adherence (94.6%), patients’ physical and psychological health amelioration (94.3%) and increased physician/nurse satisfaction (93.9%). More nurses and allied health professionals and fewer physicians and students reported that efficient health communication strategies are associated with lower costs for the healthcare system (*p* < 0.001) and a lower number of patient readmissions/revisits (*p* = 0.038). 

Overall, the following communication skills ranked among the five most crucial: the ability to listen to patients (agreement rate: 80.6%), to deliver unfortunate news regarding a diagnosis (78.8%), to effectively obtain medical history (78.9%), to answer patients’ questions clearly (78.4%) and to explain in simple language a health problem or treatment plan to the patient (75.6%) (Table 3). Showing empathy towards the patients’ emotional concerns was also highly evaluated by most healthcare professionals. 

When subgroup analysis was performed according to the participant’s profession, a different ranking was observed. Physicians considered the significance of effectively obtaining medical history as more important than other professions. On the other hand, physicians evaluated the following communications skills as less important compared to nurses (all *p* < 0.05): handle patient anger/disappointment/fear, assess the patient’s disease and treatment complications and react accordingly, explore the patient’s concerns and effectively prompt questions, discuss practical difficulties regarding the treatment plan and effectively include patients in decision-making. A lower percentage of students (61.5%) assessed the ability to listen to patients as very important compared to the other groups (*p* < 0.001).

### 3.4. Barriers to Effective Health Communication

Health professionals were also asked to evaluate potential barriers to efficient health communication, across different healthcare settings. The results are summarized in Table 4. In all healthcare settings, physicians’ sex was considered the least important barrier, followed by the physicians’ younger or older age (average score less than 4.00, in all setting for both barriers).

The barriers perceived as the most crucial ones (all received a total score higher than 3.95/5.00), in the primary healthcare and hospital settings, were the following: emotional state of patients, time restrictions, low patient health literacy, language issues, large number of patients/heavy workload/exhaustion, lack of professionals’ training in health communication skills, lack of interest from the physicians’/nurses’ part and lack of interest from the administration. In the private sector, the barriers mentioned above were considered slightly less critical than in the other sectors.

In primary healthcare, the emotional state of patients was evaluated as the most crucial barrier for successful communication by nurses, the large number of patients by physicians and students and the lack of professionals’ training in health communication skills by allied health professionals (*p* < 0.001). 

In hospitals, all health professionals assessed the emotional state and large number of patients as the two most critical barriers. Nurses assessed the “lack of professionals’ training in health communication skills” and “lack of interest on the physicians’/nurses’ part” as significantly more critical compared to physician ratings (*p* < 0.001). 

In the private practice, all participants reported the emotional state of patients as the most crucial barrier. The lack of professionals’ training in health communication skills was also reported as a common significant barrier by nurses, students and allied health professionals, but less significant among physicians in private practice (*p* < 0.001).

### 3.5. Facilitators of Effective Health Communication

The most important facilitators for effective communication (score >4.00, across all settings and all professions) were the following: training of physicians/nurses in health communication skills, an interest in health communication from higher administration, longer consultation hours/fewer patients, informed patients and the presence of cultural/language mediators (Table 5). Satisfactory remuneration of the physician or nurse was also considered an important facilitator (scores ranged from 3.82 to 3.92/5.00, across the three settings).

In primary healthcare settings, some facilitators were perceived as more critical by nurses compared with physicians (*p* < 0.05), namely the training of physicians/nurses in health communication skills, interest in health communication from higher administration and informed patients.

In hospitals, training of physicians/nurses in health communication skills was reported as more important by nurses and students when compared to physicians (*p* < 0.05). Nurses considered informed patients to be a more critical facilitator in effective communication compared to physicians (*p* < 0.001).

### 3.6. Predictors of Having Received HCT

The results from the logistic models investigating the effect of healthcare professionals’ characteristics on the likelihood of having received an HCT program in the past are presented in Table 6. Nurses were more likely to have received HCT in the past compared to physicians (OR = 1.84; 95% CI 1.16, 2.91; adjusted model). Having 6–10 years of professional experience (OR = 0.52; 95% CI 0.27, 0.99; adjusted model) indicated a lower odds of having received an HCT program in the past compared with participants with <5 years of professional experience. Living in Cyprus, Germany, Italy, Poland and Spain indicated higher odds of having participated in a training program in the past in comparison with Greece in the final model after backward selection.

## 4. Discussion

To our knowledge, there are only a few studies in the literature that address the outcomes, barriers and facilitators of effective communication from the perspective of European health professionals and the importance of different communication skills. Even though most healthcare professionals in our study highly value HCT and are willing to participate in the relevant training, more than four in ten have never received health communication training with nurses being twice as likely to have received HCT in the past. Furthermore, healthcare professionals acknowledged the contribution of yielding communication in improving professional–patient relationships, patients’ and professionals’ satisfaction and patient trust, medication adherence and physical and emotional health amelioration. More than four out of five rated listening to patients, delivering unfavorable messages, answering questions clearly and explaining problems and treatment plans in simple language as very important. Nurses overall valued health communication skills highly and, compared to physicians, considered the lack of training or interest in health communication by health professionals/administration as more critical. The emotional state of patients was perceived to be an important barrier to health communication across all healthcare settings. In contrast, time restriction and the high number of patients were considered more significant barriers in primary/hospital settings than in private practice.

The majority of healthcare professionals in our study acknowledged the contribution of effective communication to the health status, patient–physician/nurse relationship, patient and professional satisfaction and treatment adherence [2,3,4,5,6]. However, although health communication can substantially limit expenses and reduce readmission rates [3,20], we found indications that healthcare professionals, especially physicians, underestimated these benefits. Furthermore, the literature suggests that listening to the patient empathetically and adapting the language to the patient’s understanding and educational level can be essential factors in increasing a patient’s trust and satisfaction [21,22,23], factors understood by the majority of our sample as well. 

Differences in the perceived importance of communication skills and the need for HCT were also evident between professions, especially among physicians and nurses. Our results show that physicians probably tend to underestimate the contribution of various communication skills in efficient communication more than nurses, especially around the patient’s emotional management, disease/treatment comprehension by patients and the inclusion of the patient in shared decision-making. A previous study has shown that nurses believe empathy is a necessary therapeutic tool in providing care, while physicians reported more often that their understanding of the patient’s feelings does not influence their treatment [24]. Nevertheless, in both the aforementioned and the current study, disparities in the perceived favorable outcomes of showing empathy overall were not evident. This is suggestive of the awareness gap regarding the use of practices requiring empathy and the perception of implementing empathy among physicians. To further reinforce this theory, we should account for the following: most physicians consider themselves adequately equipped with health communication skills [12], overestimate their abilities to communicate with patients [11] and may often prioritize clinical expertise, outcomes and evidence-based therapies, instead of communicating with the patient [3,21,25]. Such findings are evident in another study, where most orthopedic surgeons believed they had communicated successfully, while most of their patients disagreed [26]. On the other hand, in their everyday practice, nurses tend to be more involved with the patient’s daily life and have to communicate with the patient more frequently [27]. This continuous communication requires constant empathy and assessment of the patient’s emotional state [27,28], although it may not always be efficient due to multiple barriers [29].

Most of the reported perceived barriers and facilitators in health communication in our study are common findings in the literature, primarily through qualitative studies that examine the patient–physician relationship from both sides. These barriers and facilitators include the increased number of patients/high workload, time restrictions, patients’ mental state, language, health literacy and lack of communication skills among professionals [11,19,30,31,32,33,34,35,36,37,38,39]. However, most of these studies do not examine how these barriers are understood by professionals, highlighting the novelty of the current study. Healthcare professionals’ sex was perceived as the least important barrier in health communication in our study, even though sex incompatibility (e.g., male patient and female physician) is often considered a substantial barrier by the patients [33,40]. Furthermore, differences between the professions’ perceived barriers and facilitators were evident. Nurses overall identified the patient’s uninformed status, emotional state and low literacy and lack of a professional’s interest in the interpersonal healthcare professional–patient relationship or training in health communication as more significant barriers than physicians. Such findings further reinforce the previous hypothesis that nurses value empathy highly in interpersonal communication with patients and consider the lack of HCT a substantial barrier [27], while physicians may focus more on the effectiveness of therapy. Further emphasizing this phenomenon, we also found that nurses are twice as likely as physicians to have actively sought and received HCT.

Our findings indicate that about half of physicians and many allied healthcare professionals have not received HCT. As confirmed in the literature, professionals in European medical settings have been inadequately trained in health communication for many years [41,42], a situation that led many European countries to devote financial resources to promote HCT [43,44], without evident beneficial results. In the last decades, a more patient-centered care model that emphasizes communication and shared decision-making has effectively replaced the passive information-based model in consultation practice [11,13,14]. Many European countries have recently shifted focus to an efficient person-centered care, enjoying a variety of benefits from better health outcomes and reduced expenses. Our results have indicated that younger professionals with less working experience or students from healthcare-related sectors may be more likely to have received HCT, although there is uncertainty around the results regarding students, due to the limited sample size. Nevertheless, many healthcare professionals have still not received adequate training to support this shift in healthcare practices, as many gaps in effective care and communication are evident [3,45]. 

A cross-sectional survey conducted a few years prior to our study with patients from 34 countries, including many European Member States, concluded that all primary healthcare systems indicated the limited potential to improve communication practices [46]. The already poor physician–patient communication was further aggravated during the COVID-19 pandemic. The high workload has led many professionals to devote even less time to patients’ care and has exacerbated work-related exhaustion and consequent professionals’ burnout [47]. Developing impactful strategies to resolve professionals’ moral distress, improving the provider’s empathy and communication skills and introducing novel and targeted communication HCT resources, may sufficiently bridge this gap of inadequate communication [47]. 

It should be acknowledged that the data collection took place in 2016–2017. While some may perceive this as dated, it still provides valuable insights within the realm of interpersonal health communication, particularly in the post-COVID-19 pandemic era. The pandemic highlighted deficiencies in health communication and revealed numerous barriers faced by healthcare professionals when conveying their advice [48]. They encountered difficulties in establishing the authenticity and reliability of their medical communication, which resulted in information uncertainty and even information overload as attempts were made to address this uncertainty [49]. Furthermore, telehealth services emerged as substitutes for in-person communication, finding many healthcare professionals unprepared to tackle the associated challenges [50]. Our article sheds light on the reasons why certain professionals lacked essential skills and were unable to deliver effective care or effectively convey their messages. In this post-pandemic era, policymakers should capitalize on elevated health communication awareness and provide adequate opportunities for HCT to address the communication gaps between professionals and patients. 

Apart from the dated data collection, the following limitations have also been identified in this study. Due to the limited sample of physicians’ and nurses’ specializations and allied health professionals’ occupations, findings were not classified for different specializations/professions. The distribution of all categories of healthcare professionals and students varied significantly between countries, preventing us from having a representative sample for each country. In addition, the sample of students was small, limiting the credibility of the findings around students. Future research should focus on identifying a larger sample of all healthcare professions, to identify disparities in the perceptions of various healthcare professionals, other than physicians and nurses. Since all the participating countries have differences in the organization and services provided by their healthcare systems, it may be impossible to generalize our findings to all healthcare settings. Moreover, the translation of the questionnaire to all consortium languages did not follow an established method to validate the translations. However, to minimize translation bias, only professionals in the field of health communication were involved in the translation. Finally, it is important to understand that the goal of this study was to understand the benefits, barriers and outcomes of effective health communication from the point of view of healthcare professionals. Such benefits and barriers are evident in the literature, but there is scarce evidence on how health professionals understand their importance.

## 5. Conclusions

Many healthcare professionals have yet to receive HCT, even though almost everyone identifies the beneficial effect of successful health communication on improved professional–patient relations, professional satisfaction and patient satisfaction, trust and medication adherence. Physicians appear to be less concerned about communication skills, the barriers and facilitators of effective communication and perceived outcomes of successful communication. Healthcare professionals should be sufficiently trained in health communication to achieve a better relationship, better prognosis and treatment results among patients. It is necessary to increase understanding of the benefits and systemic barriers to efficient communication so as to intervene also at the organizational level. Healthcare professionals must realize the importance of HCT in their everyday practice and governmental policies should focus on including HCT in the core of every health-related training program, especially at the undergraduate level.

## Figures and Tables

**Table 1:** Descriptive characteristics of healthcare professionals that participated in the H-Com project online survey.

Participants Characteristics	Total Sample (*n* = 691)	Physicians (*n* = 311)	Nurses (*n* = 257)	Students (*n* = 39)	Allied Health Professionals (*n* = 84)	*p*-Value
Country of current employment (*n* (%))						
Cyprus	26 (3.8)	6 (1.7)	20 (7.6)	0 (0.0)	1 (1.3)	<0.001
Germany	99 (14.3)	55 (17.8)	12 (4.8)	23 (58.3)	15 (17.5)	
Greece	146 (21.2)	90 (28.9)	34 (13.2)	3 (8.3)	18 (21.3)	
Italy	106 (15.3)	60 (19.5)	14 (5.6)	0 (0.0)	29 (35.0)	
Poland	92 (13.3)	60 (19.5)	26 (10)	0 (0.0)	4 (5.0)	
Portugal	64 (9.2)	0 (0.0)	61 (23.6)	0 (0.0)	1 (1.3)	
Spain	105 (15.2)	16 (5.0)	74 (28.8)	11 (29.2)	5 (6.3)	
Other	53 (7.7)	24 (7.7)	16 (6.4)	2 (4.2)	11 (12.5)	
Sex (*n* (%); Male)	250 (36.2)	153 (49.2)	67 (26.1)	11 (28.2)	19 (22.6)	<0.001
Age category (*n* (%))						
18–24 years old	59 (8.5)	11 (3.5)	16 (6.1)	29 (74.4)	3 (3.6)	<0.001
25–34 years old	127 (18.4)	44 (14.1)	59 (23.0)	9 (23.1)	15 (17.9)	
35–44 years old	166 (24.0)	78 (25.1)	67 (26.1)	0 (0.0)	21 (25.0)	
45–54 years old	187 (27.1)	84 (27.0)	78 (30.4)	1 (2.5)	24 (28.6)	
55–64 years old	135 (19.5)	81 (26.0)	35 (13.6)	0 (0.0)	19 (22.6)	
>65 years old	17 (2.5)	13 (4.3)	2 (0.8)	0 (0.0)	2 (2.5)	
Highest educational degree attained (*n* (%))						
Secondary school	49 (7.1)	13 (4.2)	10 (3.8)	23 (59.0)	2 (2.4)	<0.001
Vocational training	41 (5.9)	2 (0.3)	21 (8.1)	10 (25.6)	9 (10.7)	
Undergraduate degree (BSc)	165 (23.9)	44 (14.3)	89 (34.6)	5 (12.8)	29 (34.5)	
Graduate degree (MSc)	255 (36.9)	119 (38.4)	111 (43.2)	1 (2.6)	25 (29.8)	
Doctoral training (PhD)	181 (26.2)	133 (42.8)	26 (10.3)	0 (0.0)	19 (22.6)	
Years of professional experience (*n* (%))						
<5 years	150 (21.6)	57 (18.1)	43 (16.9)	36 (91.2)	17 (20.2)	<0.001
6–10 years	104 (15.0)	49 (15.9)	38 (14.6)	3 (8.8)	13 (15.5)	
11–20 years	165 (23.9)	81 (26.2)	62 (24.0)	0 (0.0)	21 (25.0)	
21–30 years	155 (22.5)	71 (22.7)	72 (28.0)	0 (0.0)	12 (14.3)	
>30 years	117 (17.0)	53 (17.1)	42 (16.5)	0 (0.0)	21 (25.0)	

Cells represent the count and proportion of the total column sample. *p*-values were obtained using Pearson chi-square test. Allied health professionals: psychologists, paramedics, health administration staff, nursing assistants, social workers, dentists/dental hygienists, dietitians/nutritionists, health promotion specialists, public health specialists, etc.

**Table 2:** Health professionals’ willingness to participate in HCT and perceptions regarding its need and availability of HCT.

	Total Sample (*n* = 691)	Physicians (*n* = 311)	Nurses (*n* = 257)	Students (*n* = 39)	Allied Health Professionals (*n* = 84)	*p*-Value
Necessity of specialized HCT for:						
Physicians (*n* (%))						
Necessary	618 (89.4)	278 (89.4)	228 (88.6)	38 (97.4)	74 (88.1)	0.799
Good to have but not necessary	68 (9.9)	31 (10.0)	27 (10.6)	1 (2.6)	9 (10.7)	
Unnecessary	5 (0.7)	2 (0.6)	2 (0.8)	0 (0.0)	1 (1.2)	
Nurses (*n* (%))						
Necessary	616 (89.2)	274 (88.2)	236 (91.8)	37 (94.9)	69 (81.9)	0.154
Good to have but not necessary	72 (10.4)	35 (11.1)	21 (8.2)	2 (5.1)	14 (16.9)	
Unnecessary	3 (0.4)	2 (0.7)	0 (0.0)	0 (0.0)	1 (1.2)	
HC is considered more important in (*n* (%))						
Primary healthcare	61 (8.8)	26 (8.4)	27 (10.5)	2 (5.1)	6 (7.2)	0.193
Hospital	28 (4.1)	14 (4.5)	11 (4.3)	1 (2.6)	2 (2.4)	
Private practice	6 (0.9)	4 (1.3)	0 (0.0)	2 (5.1)	0 (0.0)	
All of the above settings	593 (85.8)	266 (85.5)	218 (84.8)	34 (87.2)	75 (89.2)	
Other setting	3 (0.4)	1 (0.3)	1 (0.4)	0 (0.0)	1 (1.2)	
Receipt of HCT in the past (*n* (%); Yes)	394 (57.0)	152 (48.9)	168 (65.4)	27 (69.2)	47 (55.4)	<0.001
Educational level at which HCT was received (*n* (% of those who have received HCT))						
Graduate level	202 (51.3)	93 (61.2)	91 (59.9)	16 (10.5)	2 (1.3)	<0.001
Postgraduate level	274 (69.5)	106 (69.7)	106 (69.7)	35 (23)	27 (17.8)	
Residency training	339 (86.0)	120 (78.9)	151 (99.3)	41 (27)	27 (17.8)	
Continuous education training	230 (58.4)	101 (66.4)	75 (49.3)	28 (18.4)	26 (17.1)	
Other	361 (91.6)	143 (94.1)	156 (102.6)	36 (23.7)	26 (17.1)	
Willingness to participate in HCT (*n* (%); Yes)	609 (88.1)	266 (85.6)	233 (90.6)	33 (84.6)	77 (91.1)	0.218

HC: health communication, HCT: health communication training. *p*-values were obtained using Pearson chi-square test. Allied health professionals: psychologists, paramedics, health administration staff, nursing assistants, social workers, dentists/dental hygienists, dietitians/nutritionists, health promotion specialists, public health specialists, etc.

**Table 3:** Healthcare professionals’ perceptions regarding the beneficial impact of effective health communication and the importance of various communication skills (*n* = 691; online survey of the H-Com project).

	Total Sample (*n* = 691)	Physicians (*n* = 311)	Nurses (*n* = 257)	Students (*n* = 39)	Allied Health Professionals (*n* = 84)	*p*-Value
Effective health communication contributes to (% Agree/Strongly agree):						
Improved physician/nurse–patient relations	97.5	97.4	97.6	97.5	97.6	0.267
Increased patient satisfaction	97.4	97.1	98.1	94.8	97.5	0.014
The improvement of patient trust to healthcare professionals	96.6	95.1	98.4	94.9	97.5	0.029
Patient medication adherence	94.6	94.2	94.5	97.4	95.2	0.506
The improvement of patients’ physical and psychological health	94.3	92.8	95.3	94.9	96.4	0.019
Increased physician/nurse satisfaction	93.9	93.8	95.2	94.8	89.9	0.255
Fewer incidents of medical malpractice	81.3	77.0	85.1	81.6	85.3	0.442
Lower costs for the healthcare system	78.5	72.3	85.6	71.8	83.1	<0.001
A lower number of patient admissions/re visits	74.8	72.0	79.8	56.4	78.5	0.038
Importance of communication skills (% Very important):						
Effectively obtain medical history	78.6	84.8	75.8	69.2	68.3	<0.001
Listen to your patient	80.6	78.7	87.2	61.5	76.5	0.003
Tell/give bad news to the patient	78.8	77.2	84.2	73.7	70.4	0.117
Answer the patient’s questions clearly	78.4	77.0	81.6	74.4	75.6	0.623
Explain in layman’s terms a health problem or treatment plan to the patient	75.6	72.0	83.8	69.2	66.7	0.031
Assess the patient’s understanding of their disease and treatment plan and react accordingly	73.2	70.1	80.6	69.2	64.2	0.023
Handle patient anger/disappointment/fear	72.6	71.2	79.4	51.3	67.1	0.008
Show empathy towards the patient’s emotional concerns	70.2	67.5	77.3	56.4	64.6	0.145
Clearly describe a medical situation/treatment plan to patients with low health literacy	69.6	65.4	76.7	66.7	65.4	0.154
Explore the patient’s concerns and effectively prompt questions	68.1	66.2	76.5	55.3	55.6	0.005
Discuss the practical difficulties of the patient’s treatment plan	66.5	64.0	73.0	51.3	63.4	0.018
Effectively include patients in decision-making regarding their treatment plan	66.0	60.3	76.7	51.3	61.3	0.004

*p*-value is based on Pearson chi-squared test. Allied health professionals: psychologists, paramedics, health administration staff, nursing assistants, social workers, dentists/dental hygienists, dietitians/nutritionists, health promotion specialists, public health specialists, etc.

**Table 4:** Healthcare professionals’ perceptions regarding the barriers they confront in terms of effective health communication in different healthcare settings (primary healthcare, hospitals and private practice), for the total sample and separately for each medical specialty (online survey of the H-Com project).

	Barriers of Effective Health Communication in: (Mean (SD))	Total Sample (*n* = 691)	Physicians (*n* = 311)	Nurses (*n* = 257)	Students (*n* = 39)	Allied Health Professionals (*n* = 84)	*p*-Value
Primary healthcare	Emotional state of patients	4.32 (0.78)	4.21 (0.81)	4.50 (0.62)	4.38 (0.75)	4.11 (0.98)	<0.001
	Time restrictions	4.34 (0.81)	4.38 (0.83)	4.40 (0.73)	4.31 (0.86)	3.99 (0.88)	<0.001
	Low health literacy of patients	3.98 (0.89)	3.91 (0.88)	4.10 (0.85)	3.74 (0.97)	4.00 (1.01)	0.022
	Language issues (e.g., patients from migrant backgrounds)	4.27 (0.91)	4.17 (1.02)	4.39 (0.79)	4.13 (0.84)	4.30 (0.80)	0.026
	Large number of patients/heavy workload/exhaustion	4.43 (0.73)	4.47 (0.72)	4.43 (0.72)	4.44 (0.68)	4.28 (0.83)	0.196
	Lack of professionals’ training in health communication skills	4.23 (0.87)	4.07 (0.91)	4.41 (0.73)	3.97 (1.05)	4.35 (0.90)	<0.001
	Lack of interest on the physicians’/nurses’ part	4.19 (0.94)	4.04 (0.99)	4.36 (0.85)	4.23 (0.96)	4.27 (0.93)	0.001
	Lack of interest from administration	4.09 (1.02)	3.99 (1.08)	4.24 (0.93)	3.87 (1.22)	4.16 (0.88)	0.015
	Physicians’ older age	2.96 (1.33)	2.84 (1.35)	3.07 (1.27)	2.85 (1.42)	3.18 (1.34)	0.073
	Physicians’ younger age	2.82 (1.22)	2.62 (1.21)	3.01 (1.16)	2.54 (1.23)	3.09 (1.28)	<0.001
	Physicians’ sex	2.23 (1.28)	2.00 (1.21)	2.41 (1.26)	1.92 (1.18)	2.65 (1.42)	<0.001
	Problems with salaries of the physician or nurse	3.20 (1.31)	3.15 (1.33)	3.32 (1.27)	3.05 (1.19)	3.06 (1.36)	0.253
Hospitals	Emotional state of patients	4.40 (0.75)	4.35 (0.76)	4.50 (0.69)	4.42 (0.68)	4.29 (0.89)	0.056
	Time restrictions	4.37 (0.85)	4.31 (0.91)	4.50 (0.75)	4.39 (0.79)	4.16 (0.88)	0.008
	Low health literacy of patients	3.96 (0.95)	3.85 (0.97)	4.11 (0.90)	3.84 (0.95)	3.99 (0.95)	0.013
	Language issues (e.g., patients from migrant backgrounds)	4.23 (0.90)	4.15 (0.98)	4.31 (0.86)	4.21 (0.81)	4.33 (0.76)	0.159
	Large number of patients/heavy workload/exhaustion	4.43 (0.77)	4.39 (0.85)	4.52 (0.66)	4.38 (0.76)	4.36 (0.73)	0.155
	Lack of professionals’ training in health communication skills	4.18 (0.91)	3.99 (1.02)	4.38 (0.73)	4.08 (1.05)	4.33 (0.75)	<0.001
	Lack of interest on the physicians’/nurses’ part	4.08 (1.02)	3.89 (1.09)	4.26 (0.92)	4.13 (1.02)	4.23 (0.90)	<0.001
	Lack of interest from administration	3.96 (1.11)	3.87 (1.18)	4.07 (1.03)	3.76 (1.16)	4.06 (0.97)	0.100
	Physicians’ older age	2.83 (1.32)	2.72 (1.32)	2.91 (1.30)	2.61 (1.33)	3.05 (1.35)	0.102
	Physicians’ younger age	2.63 (1.24)	2.41 (1.20)	2.79 (1.22)	2.47 (1.27)	3.03 (1.30)	<0.001
	Physicians’ sex	2.19 (1.28)	2.00 (1.23)	2.35 (1.29)	1.76 (1.13)	2.63 (1.34)	<0.001
	Problems with salaries of the physician or nurse	3.26 (1.32)	3.15 (1.38)	3.44 (1.24)	3.08 (1.28)	3.22 (1.29)	0.053
Private practice	Emotional state of patients	4.29 (0.87)	4.25 (0.92)	4.36 (0.81)	4.36 (0.71)	4.22 (0.92)	0.369
	Time restrictions	3.74 (1.21)	3.54 (1.27)	3.94 (1.13)	3.89 (1.10)	3.76 (1.15)	0.002
	Low health literacy of patients	3.86 (1.05)	3.73 (1.10)	3.96 (1.02)	3.75 (1.13)	4.05 (0.86)	0.028
	Language issues (e.g., patients from migrant backgrounds)	4.03 (1.03)	3.96 (1.09)	4.08 (1.00)	3.95 (1.00)	4.18 (0.91)	0.284
	Large number of patients/heavy workload/exhaustion	3.69 (1.23)	3.43 (1.28)	3.86 (1.20)	3.92 (0.98)	3.97 (1.06)	<0.001
	Lack of professionals’ training in health communication skills	4.06 (0.97)	3.88 (1.03)	4.22 (0.85)	4.14 (0.92)	4.22 (1.02)	<0.001
	Lack of interest on the physicians’/nurses’ part	3.81 (1.18)	3.57 (1.25)	4.00 (1.09)	3.94 (1.01)	4.04 (1.11)	<0.001
	Lack of interest from administration	3.43 (1.40)	3.16 (1.45)	3.68 (1.30)	3.17 (1.44)	3.78 (1.27)	<0.001
	Physicians’ older age	2.68 (1.32)	2.51 (1.32)	2.82 (1.26)	2.54 (1.41)	2.97 (1.36)	0.009
	Physicians’ younger age	2.64 (1.25)	2.45 (1.23)	2.78 (1.19)	2.41 (1.28)	3.00 (1.36)	0.001
	Physicians’ sex	2.17 (1.29)	1.94 (1.22)	2.35 (1.27)	1.81 (1.22)	2.62 (1.42)	<0.001
	Problems with salaries of the physician or nurse	3.01 (1.38)	2.87 (1.39)	3.23 (1.37)	2.68 (1.27)	3.00 (1.37)	0.013

SD = standard deviation. Each barrier was scored on a scale 1 to 5 (1 = low, 5 = high importance). The mean and the standard deviation are presented in the form of mean (SD). *p*-value is based on univariate analysis of variance (ANOVA). Allied health professionals: psychologists, paramedics, health administration staff, nursing assistants, social workers, dentists/dental hygienists, dietitians/nutritionists, health promotion specialists, public health specialists, etc.

**Table 5:** Healthcare professionals’ perceptions regarding the facilitators of health communication in different healthcare settings (primary healthcare, hospitals and private practice), for the total sample and separately for each medical specialty (*n* = 691; online survey of the H-Com project).

	Facilitators of Health Communication in (mean (SD)):	Total Sample (*n* = 691)	Physicians (*n* = 311)	Nurses (*n* = 257)	Students (*n* = 39)	Allied Health Professionals (*n* = 84)	*p*-Value
Primary healthcare	Training of physicians/nurses in health communication skills	4.56 (0.70)	4.44 (0.81)	4.71 (0.51)	4.54 (0.68)	4.52 (0.74)	<0.001
	Interest in health communication from higher administration	4.35 (0.83)	4.28 (0.91)	4.50 (0.67)	4.10 (1.05)	4.28 (0.83)	0.002
	Longer consultation hours/fewer patients	4.49 (0.72)	4.50 (0.74)	4.55 (0.66)	4.41 (0.82)	4.33 (0.73)	0.088
	Informed patients	4.22 (0.90)	4.11 (0.95)	4.38 (0.84)	4.03 (0.96)	4.17 (0.83)	0.002
	Presence of cultural/language mediators	4.23 (0.90)	4.16 (0.98)	4.27 (0.84)	4.36 (0.71)	4.26 (0.86)	0.385
	Satisfactory remuneration of the physician or nurse	3.92 (1.13)	3.94 (1.16)	3.98 (1.08)	3.77 (1.22)	3.70 (1.12)	0.190
Hospitals	Training of physicians/nurses in health communication skills	4.60 (0.68)	4.50 (0.78)	4.73 (0.49)	4.64 (0.63)	4.53 (0.75)	0.001
	Interest in health communication from higher administration	4.39 (0.82)	4.37 (0.89)	4.50 (0.70)	4.10 (0.97)	4.28 (0.91)	0.011
	Longer consultation hours/fewer patients	4.47 (0.73)	4.46 (0.76)	4.59 (0.62)	4.44 (0.75)	4.19 (0.87)	<0.001
	Informed patients	4.20 (0.89)	4.04 (0.97)	4.43 (0.74)	4.08 (0.93)	4.12 (0.87)	<0.001
	Presence of cultural/language mediators	4.29 (0.88)	4.21 (0.94)	4.33 (0.83)	4.36 (0.78)	4.29 (0.81)	0.420
	Satisfactory remuneration of the physician or nurse	3.95 (1.12)	3.95 (1.14)	4.04 (1.07)	3.82 (1.12)	3.73 (1.20)	0.170
Private practice	Training of physicians/nurses in health communication skills	4.50 (0.77)	4.41 (0.86)	4.64 (0.61)	4.53 (0.73)	4.44 (0.88)	0.007
	Interest in health communication from higher administration	4.03 (1.19)	3.88 (1.31)	4.29 (0.97)	3.71 (1.39)	3.95 (1.13)	<0.001
	Longer consultation hours/fewer patients	4.15 (0.95)	4.06 (1.00)	4.28 (0.88)	4.13 (1.02)	4.10 (0.89)	0.073
	Informed patients	4.14 (0.92)	4.06 (0.95)	4.28 (0.85)	4.08 (1.00)	4.05 (0.91)	0.035
	Presence of cultural/language mediators	4.09 (1.00)	3.98 (1.10)	4.18 (0.92)	4.13 (0.96)	4.16 (0.86)	0.125
	Satisfactory remuneration of the physician or nurse	3.82 (1.21)	3.75 (1.29)	4.00 (1.08)	3.54 (1.22)	3.65 (1.21)	0.017

SD = standard deviation. Each facilitator was scored on a scale 1 to 5 (1 = low, 5 = high importance). The mean and the standard deviation are presented in the form of mean (SD). *p*-value is based on univariate analysis of variance (ANOVA). Allied health professionals: psychologists, paramedics, health administration staff, nursing assistants, social workers, dentists/dental hygienists, dietitians/nutritionists, health promotion specialists, public health specialists, etc.

**Table 6:** The effect of healthcare professionals’ characteristics on the likelihood of having received HCT in the past, following logistic regression models.

Healthcare Professionals’ Characteristics	Univariate Analysis (Unadjusted Models) OR (95% CI)	Multi-Adjusted Model OR (95% CI)
Sex (ref: female)		
Male	0.79 (0.58, 1.08)	0.89 (0.62, 1.28)
Professional status (ref: physicians)		
Nurses	1.98 (1.40, 2.77) ***	1.84 (1.16, 2.91) **
Students	2.35 (1.15, 4.81) **	1.98 (0.59, 6.63)
Other	1.30 (0.80, 2.12)	1.19 (0.69, 2.04)
Country of current employment (ref: Greece)		
Germany	3.21 (1.85, 5.57) ***	3.12 (1.73, 5.62) ***
Cyprus	4.12 (1.61, 10.54) **	3.18 (1.16, 8.68) **
Italy	1.74 (1.03, 2.93) **	1.63 (0.92, 2.86) *
Poland	1.72 (0.99, 2.96) *	1.70 (0.96, 3.02) *
Portugal	2.77 (1.48, 5.19) **	1.70 (0.83, 3.46)
Spain	3.16 (1.84, 5.43) ***	2.08 (1.14, 3.77) **
Other	3.11 (1.58, 6.13) **	3.09 (1.53, 6.24) **
Age category (ref: 18–24 years old)		
25–34 years old	0.57 (0.29, 1.11) *	0.99 (0.34, 2.92)
35–44 years old	0.46 (0.24, 0.89) **	0.93 (0.28, 3.05)
45–54 years old	0.38 (0.20, 0.71) **	0.55 (0.16, 1.95)
55–64 years old	0.53 (0.27, 10.2) *	0.62 (0.16, 2.44)
>65 years old	0.42 (0.14, 1.27)	0.38 (0.07, 2.23)
Highest educational degree attained (ref: secondary school)		
Vocational training	0.45 (0.18, 1.08) *	0.45 (0.13, 1.74)
Undergraduate degree (BSc)	0.51 (0.26, 1.03) *	1.12 (0.32, 3.92)
Graduate degree (MSc)	0.54 (0.28, 1.07) *	1.57 (0.46, 5.42)
Doctoral training (PhD)	0.52 (0.26, 1.04) *	2.19 (0.61, 7.80)
Years of professional experience (ref: ≤5 years)		
6–10 years	0.41 (0.25, 0.70) **	0.52 (0.27, 0.99) **
11–20 years	0.59 (0.37, 0.93) **	0.87 (0.43, 1.78)
21–30 years	0.63 (0.39, 1.00) *	1.13 (0.48, 1.64)
>30 years	0.84 (0.51, 1.40)	1.70 (0.64, 4.52)

*** *p* < 0.01; ** *p* < 0.05; * *p* < 0.10. Abbreviations: Odds Ratio (OR); 95% Confidence Interval (95% CI), HCT: health communication training, ref: reference group. Multi-adjusted model has the following variables as adjustments: sex (females as reference category), professional status (physicians as reference category), country of current employment (Greece as reference category), age category (“18–24 years old” as reference category), highest educational degree attained (secondary school as reference category) and years of professional experience (“<5 years” as reference category).

## Data Availability

The data that support the findings of this study are available from the corresponding author (DVD), upon reasonable request.
